# Carbon SH-SAW-Based Electronic Nose to Discriminate and Classify Sub-ppm NO_2_

**DOI:** 10.3390/s22031261

**Published:** 2022-02-07

**Authors:** Carlos Cruz, Daniel Matatagui, Cristina Ramírez, Isidro Badillo-Ramirez, Emmanuel de la O-Cuevas, José M. Saniger, Mari Carmen Horrillo

**Affiliations:** 1 Grupo de Tecnología de Sensores Avanzados (SENSAVAN), Instituto de Tecnologías Físicas y de la Información (ITEFI), CSIC, 28006 Madrid, Spain; carmen.horrillo.guemes@csic.es; 2 Department of Electronics, University of Alcala, 28871 Alcala de Henares, Madrid, Spain; 3 Dpto. de Ingeniería Informática, Escuela Politécnica Superior, Universidad Autónoma de Madrid, 28049 Madrid, Spain; 4 Institute of Ceramics and Glass, ICV-CSIC, Kelsen 5, Cantoblanco, 28049 Madrid, Spain; cristina.ramirez@icv.csic.es; 5 Instituto de Ciencias Aplicadas y Tecnología, Universidad Nacional Autónoma de México, Circuito Exterior S/N, Ciudad Universitaria, Ciudad de Mexico 04510, Mexico; ibadillo@ciencias.unam.mx (I.B.-R.); emmanuel.de@correo.nucleares.unam.mx (E.d.l.O.-C.); jose.saniger@icat.unam.mx (J.M.S.); 6 Center for Intelligent Drug Delivery and Sensing Using Microcontainers and Nanomechanics (IDUN), Department of Health Technology, Technical University of Denmark, 2800 Kongens Lyngby, Denmark; 7 Unidad Académica de Física, Universidad Autónoma de Zacatecas, Zacatecas 98068, Mexico

**Keywords:** electronic nose, NO_2_, carbon nanomaterials, graphene oxide, surface acoustic wave (SAW), pollutants, discrimination, classification, Machine Learning (ML)

## Abstract

In this research, a compact electronic nose (e-nose) based on a shear horizontal surface acoustic wave (SH-SAW) sensor array is proposed for the NO_2_ detection, classification and discrimination among some of the most relevant surrounding toxic chemicals, such as carbon monoxide (CO), ammonia (NH_3_), benzene (C_6_H_6_) and acetone (C_3_H_6_O). Carbon-based nanostructured materials (CBNm), such as mesoporous carbon (MC), reduced graphene oxide (rGO), graphene oxide (GO) and polydopamine/reduced graphene oxide (PDA/rGO) are deposited as a sensitive layer with controlled spray and Langmuir–Blodgett techniques. We show the potential of the mass loading and elastic effects of the CBNm to enhance the detection, the classification and the discrimination of NO_2_ among different gases by using Machine Learning (ML) techniques (e.g., PCA, LDA and KNN). The small dimensions and low cost make this analytical system a promising candidate for the on-site discrimination of sub-ppm NO_2_.

## 1. Introduction

Chemical sensors play a relevant role in our modern society for mobile applications, traffic safety and health care. One of the principal functions of these sensors is the monitoring of chemical compounds. It has been becoming increasingly challenging in several applications related to air quality assessment [1,2,3,4,5,6,7] and medical diagnostics [8,9]. These needs have led to the emergence of new generations of low cost, portable and reliable gas sensor devices with high potential discrimination among low concentrations of analytes of interest. The combustion of fossil fuels is the major source of nitrogen oxide (NO_x_) emissions into the atmosphere. In addition, the long-term exposure to NO_2_ levels produces harmful effects for humans and living beings, which can be detected by deploying chemical sensors based on carbon material [10,11,12].

Surface acoustic wave (SAW) devices are a type of chemical sensor with high potentialities to detect low gas concentrations at room temperature (RT) due to the high sensitivity and reduced size. In addition, novel sensing applications open the possibility for custom SAW devices [13,14,15,16,17]. SAW sensors are mainly used as gravimetric (mass sensitive) transducers. Adsorption of molecules on the SAW surface produces an increase in density per unit area that modulates the acoustic wave velocity [18,19], which is most commonly measured through a shift of the oscillation frequency. In recent years, elastic sensitivity has been presented as a novel way to measure gases by a small shift in elastic properties of nanostructured layers [11,20,21,22,23]. Several studies to achieve sensors based on nanostructured sensitive layers had been performed [24,25,26], resulting in highly sensitive and selective sensors to improve electronic nose technology for detecting, discriminating and classifying target gases. For example, Bhasker et al. [27] conclude that both mass loading and change in elasticity, are the sensing mechanisms in ammonia detection. On the other hand, Yongliang et al. [28] emphasize the benefits of the elastic loading effect in the SAW sensor to a more significant response to H_2_S gas with excellent stability, selectivity and humidity resistance. For instance, SAW carbon-based sensors configuration could make it possible to operate under mass and elastic regimens [22].

Pattern recognition methods are commonly applied to the sustainable environment domain [29] and can be performed for classification and discrimination of the sensor field as an important component of the e-nose. Principal component analysis (PCA) is applied for dimensionality reduction features that contain most of the patterns for discriminative information [30,31,32,33]. Artificial neural network algorithms are widely used for pattern recognition of chemical compounds [34]. The fast training and robustness make the suggested patterns procedure an excellent classifier for sensor array data [35]. High accuracy of up to 90% confirms the effectiveness of the classification analysis, especially for the e-nose platforms [36,37]. Linear discriminant analysis (LDA) or K nearest-neighbor (KNN) are also well-known machine learning (ML) approaches for pattern recognition, able to assign class descriptors to the analytes with a high degree of confidence [38,39,40,41]. However, the system needs to be trained with known samples and the results are influenced by the selected samples tested.

In this work, we analyze the e-nose capabilities of different carbon-based sensors for selective NO_2_ classification. In addition, with the ML techniques, we perform both the extraction of distinctive information to discriminate the observed analytes and the carbon-based sensor potential for low concentration detection of NO_2_. The sensing performance of the developed e-nose operates at RT and under different exposures to analytes, achieving NO_2_ selectivity against interfering gases such as CO, NH_3_, C_6_H_6_ and C_3_H_6_O. Here, we demonstrate that the elastic and the mass loading effects of carbon-based sensors are suitable for NO_2_ discrimination by supervised and unsupervised ML techniques in a versatile and compact system.

## 2. Materials and Methods

### 2.1. Materials

Graphene oxide (GO) powder was purchased from Graphenea (San Sebastian, Spain). Dopamine (DA) hydrochloride and Tris buffer were purchased from Sigma Aldrich (St. Louis, MO, USA). Graphitized mesoporous carbon (MC) and Gum Arabic (GA) powders were purchased from Sigma Aldrich (Madrid, Spain).

### 2.2. SH-SAW Sensors Fabrication

SH-SAW was propagated on the ST-cut quartz substrate (perpendicular to the x crystallographic axis). A wave with λ = 28 μm was generated and detected by interdigital transducers (IDTs) with a double electrode configuration. The IDTs, with aluminum of 200 nm thickness, were made by RF sputtering combined with standard lithographic techniques. The distance center to center between IDTs and the acoustic aperture were 150 and 75 λ, respectively. The guided SH-SAW, well-known as the Love wave, was achieved using a 3.5 µm film of SiO_2_ grown on the piezoelectric substrate by plasma-enhanced chemical vapor deposition, obtaining a resonance frequency of around 160 MHz.

### 2.3. Sensitive Layer Fabrication

In this research, we developed an array of four sensors using the following carbon-based materials as sensitive layers:

- Mesoporous Carbon (MC). Gum Arabic (GA) was dissolved in deionized (DI) water to prepare a solution of 2 mg/mL. Then, 1 mg of MC was added to prepare a dispersion using an extended low power ultrasonication bath (Branson 5510) for 7 h. Afterwards, the dispersion was deposited on the SH-SAW sensor by spray coating in order to obtain a reproducible deposition method.
- Graphene Oxide (GO). An aqueous dispersion of GO (0.5 mg/mL) was prepared by mixing GO powder with DI water:methanol (1:5 volumetric ratio), then the mixture was sonicated for 15 min. The dispersion was used immediately for deposition using the Lagmuir–Blodgett (LB) technique.
- Reduced GO (rGO). The reduction of graphene oxide (reduced graphene oxide, rGO) was performed in a U reactor with a fritted plate 1.5 cm in diameter, where 100 mg of GO powder was heated at 400 °C for 2 h under constant flow of a 100 mL·min^−1^ H_2_/Ar (10% H_2_ balance Ar) mixture. Then, the reaction was allowed to cool until it reached RT. The synthesized product was a fine black powder. Further, a homogeneous rGO dispersion was obtained by mixing approximately 2.0 mg of rGO powder with 8 mL of methanol and sonicated for 4 h at RT.
- GO and rGO Langmuir–Blodgett film formation. Langmuir–Blodgett (LB) deposition on the sensor chip was performed in a trough KSV 5000 alternating multilayer LB system (KSV Finland). The trough was first cleaned with chloroform and then filled with ultrapure water (Milli-Q system, 18.2 MΩ cm and simplicity 185, Millipore, Burlington, MA, USA). The rGO/GO dispersion was spread onto the water surface dropwise using a glass syringe at a speed of ~100 μL/min. The surface pressure was monitored using a tensiometer attached to a Wilhelmy plate. Barriers compressed the film at a speed of 15 mm/min. The rGO monolayer was transferred to an SH-SAW sensor using the vertical lifting method at 22 °C. Z-type multilayer structures were prepared by the vertical deposition method at a dipping speed of 10 mm/min. The same procedure was followed to prepare GO monolayer substrates.
- Polydopamine (PDA) film formation on an rGO chip. PDA was obtained by dissolving DA hydrochloride powder in Tris buffer (pH 8.6) to obtain a DA concentration of 0.01 M. Then, the previously fabricated rGO (LB) sensor chip was immediately immersed in the DA/Tris solution at RT for 30 min. The PDA/rGO chip was rinsed three times with ultrapure water and dried in a dissector.

### 2.4. Structural and Morphological Characterization

Microstructural characterization of the sensitive layers was performed by field emission scanning electron microscopy (FESEM, S-4700, Hitachi, Barcelona, Spain) and Raman microscopy (Alpha 300RA, WITec GmbH, Ulm, Germany). Raman measurements were acquired with a 532 nm laser excitation wavelength with an integration time of 0.6 s and 10 accumulations; three measurements were acquired at different positions in the central region of the layer and averaged.

### 2.5. Experimental Setup for Gas Measurement

**Figure 1:**
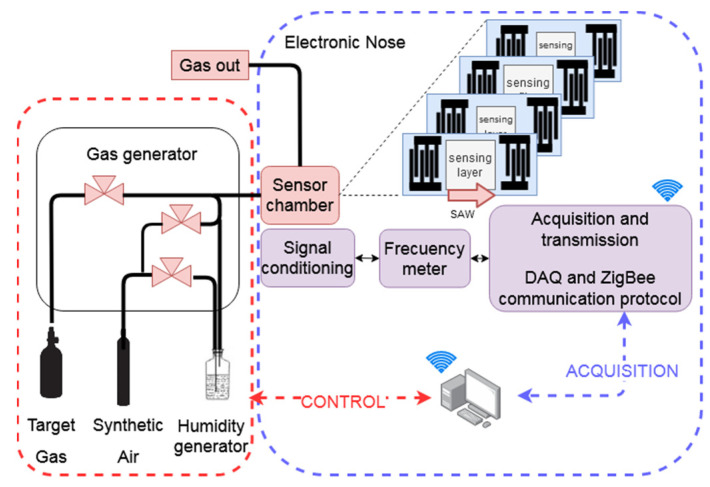
Experimental setup deployed for custom concentration measurements.

The SH-SAW sensor array was characterized by an automatic and controlled gas line for sensor characterization in groups of different gaseous environments. The gas sample generator (Figure 1) consisted of three mass flow controllers that obtain desirable concentrations regulating the flow rates of both the synthetic air and the chosen target. The process was performed by switching between a gas sample for 2 min (exposition time) and the synthetic air for 20 min (purge time) at a constant flow of 100 mL/min. Target gases were supplied by gas cylinders with a selectable concentration balanced with the carrier gas, all of them from Nippon Gases: NO_2_ (1 ppm), CO (10 ppm), NH_3_ (50 ppm), benzene (C_6_H_6_) (50 ppm) and acetone (C_3_H_6_O) (50 ppm). It was also possible to distinguish among concentrations with higher sensor responses in terms of increasing concentration steps. Then, the initial sample concentration was diluted with synthetic air to obtain lower concentrations such as 0.1–0.6 ppm for NO_2_, 1–6 ppm for CO and 10–40 ppm for NH_3_, benzene (C_6_H_6_) or acetone (C_3_H_6_O).

### 2.6. Electronic Nose Configuration

The system (e-nose) was deployed in a modular configuration:

- The signal conditioning module. The SH-SAW signals were generated using a set of microwave circuits that consisted of two amplification states and a directional coupler.
- The multiplexor module. The signal of each sensor oscillator was selected and forwarded to a single output, which was mixed with an oscillator-based reference signal, obtaining a new signal around 1 MHz. The reference device also compensates for external disturbances such as changes of temperature.
- The acquisition and transmission module. A microcontroller was used to measure the resulting signal of the multiplexor module. The e-nose was kept at controlled RT while variations of the frequency over time were recorded by wireless communication (XBEE protocol). The experiment control, the real time data acquisition and the classification analyses were implemented with a PC using the LabVIEW and the Matlab software, respectively.

## 3. Results and Discussion

### 3.1. Electrical Characterization

The standard deviation (SD) obtained for the measured SH-SAW sensor signal was 5 Hz. Considering the minimum signal as three times higher than the noise signal, the minimum detectable measurement is a frequency shift of 15 Hz.

### 3.2. Morphological and Spectroscopic Characterization of Carbon-Based Sensitive Layers

**Figure 2:**
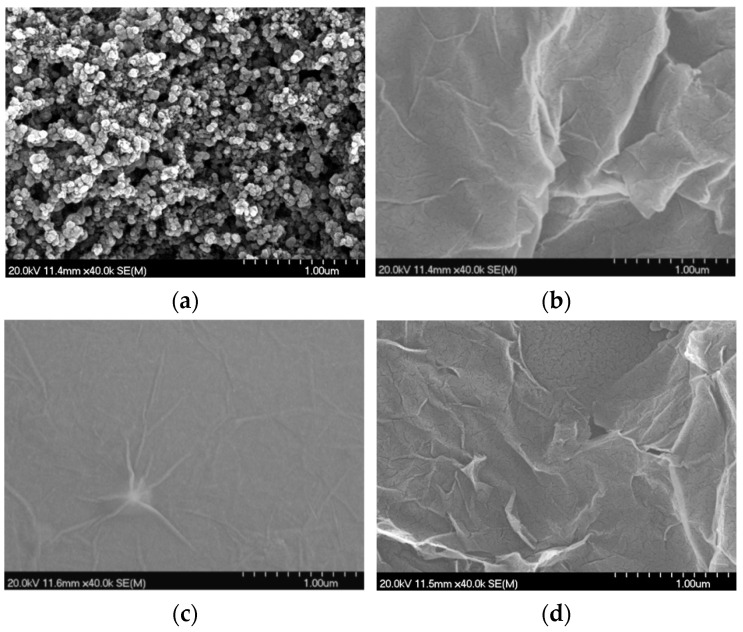
SEM images of sensitive layers based on (**a**) mesoporous carbon, (**b**) reduced graphene oxide, (**c**) graphene oxide and (**d**) polydopamine/reduced graphene oxide.

Figure 2 shows the morphology of the carbon-based materials (CBMs) deposited on the quartz substrates. A MC nanostructured layer was created by the combination of stable aqueous dispersion of the particles and air-brush spray deposition technique (Figure 2a). Particle distribution is identified with particle sizes below 100 nm and pores in the order of 100–200 nm. Figure 2b shows the GO layer, where there is deposition of completely extended sheets favored by the LB technique, showing a flat surface with wrinkles, edges and some particular bumped features of 300 nm in diameter [42]. On the other hand, Figure 2b,d shows the sensitive layers based on rGO and PDA/rGO, respectively. The thermal reduction of GO originated the crumpling of rGO sheets, though the use of a PDA treatment had a smoothening effect. It is important to notice that although the quartz surface was effectively coated due to the material’s morphology and deposition technique, the MC layer thickness is expected to be far larger than GO films.

**Figure 3:**
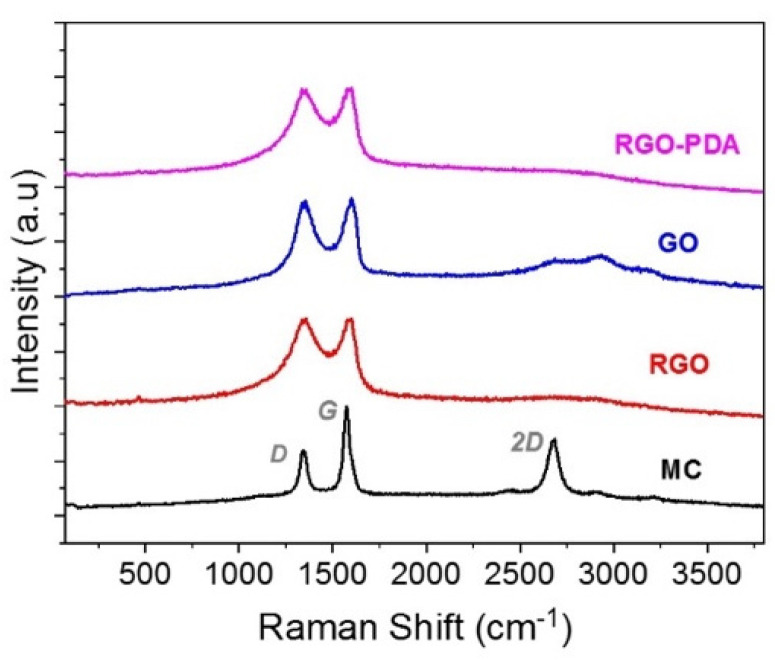
Raman spectra of different carbon-based sensitive layers.

The Raman spectrum of MC (Figure 3 black) presents the three characteristic bands of graphitized materials: D, G and 2D vibrational modes, with moderately ordered structure (I_D_/I_G_ 0.57). Similarly, GO, rGO and PDA/rGO films show broad D and G Raman bands of similar intensities, indicating the high disorder of graphitic domains obtained by the synthesis route. I_D_/I_G_ ratios of 2.9 and 3.0 were calculated for rGO and PDA/rGO, respectively, whereas GO presented approximately half of the value. This increment in disorder ratio is associated to the removal of functional groups produced by thermal reduction. In accordance with SEM observations, the tendency to form crumples by rGO could lead to an uneven substrate coating.

### 3.3. Gas Sensor Characterization

The measured gas could be detected in a complex environment surrounded by gases for different concentrations, requiring sensors with properties of sensitivity and selectivity to discriminate and correctly classify the gases. Among the most important interfering gases, for the above-cited applications where NO_2_ detection is required, are CO, NH_3_, benzene (C_6_H_6_) and acetone (C_3_H_6_O).

**Figure 4:**
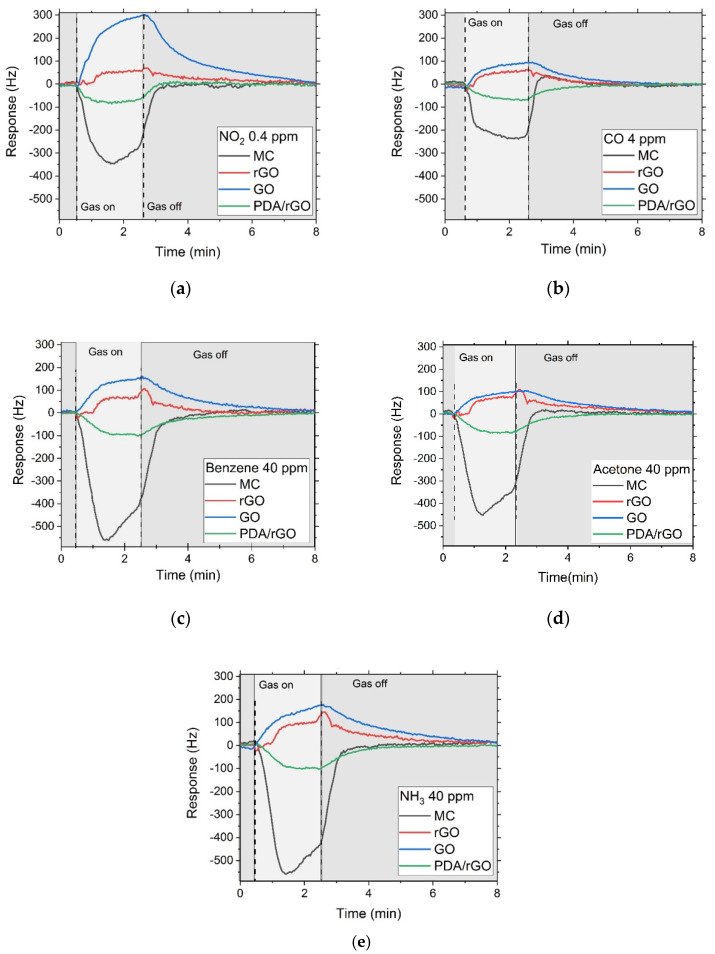
Real time response and recovery of the SH-SAW sensor array based on MC, rGO, GO and PDA/rGO for a concentration of 0.4 ppm of NO_2_ (**a**), 4 ppm of CO (**b**), 40 ppm of benzene (C_6_H_6_) (**c**), 40 ppm of acetone (C_3_H_6_O) (**d**) and 40 ppm of NH_3_ (**e**).

Experimental measurements for the gas characterization of the sensitive materials showed that sensor response is dependent on the composition of the sensitive layer. As shown in Figure 4, sensors based on MC and PDA/rGO behaved as gravimetric sensors, decreasing their frequency due to an increase in the density per unit area of the sensing layer as a consequence of the adsorbed molecules. However, the sense of the frequency shift in rGO and GO sensors was the opposite, increasing their frequency due to the change in elastic properties of the nanostructured sensitive layers caused by the interaction with gas target molecules.

The sensitive layers in the developed sensors are different in both surface chemistry and microstructure, which play a key role in the diverse sensing mechanisms that take place between the material surface and the tested gases. The MC layer contains both a large porous surface area per volume ratio and a highly ordered graphitized carbon structure, which provides a high interaction for the of gas adsorption, mainly through physisorption processes, even if chemical adsorption processes might also take part; especially for molecules with easy electronic delocalization, such as NO2, NH_3_ and C_6_H_6_. The GO layer, besides containing a large surface area, also has a high number of functional groups with oxygen due to the oxidation process of the graphene, and therefore it provides many chemical reactive sites for selective gaseous molecule adsorption which causes a large transfer of electrons, such as NO_2_ and NH_3_ [43]. Moreover, the rGO layer contains a large site of ordered aromatic groups and in addition shows a reduced number of oxygenated functional groups, in comparison with GO due to the reduction process, which reduces the chemical adsorption of many of the studied gases, but improves the π–π electrostatic interactions with aromatic molecules, such as benzene. The PDA/rGO layer contains different types of functional groups, such as aromatic, amino and hydroxy, which are linked with the reactive gas molecules, in a similar way to the GO with the oxygenated groups; however, the obtained gas adsorption performances are lower. This result might be due to the high number of unordered stacked aromatic groups and hindered reactive oxygenated groups in PDA after a thick layer formation.

The MC sensor presented a high mass response to all gases. However, the rGO and GO sensors showed an elastic response for all of them. Furthermore, the GO sensor exhibited a significant elastic response with NO_2_ gas. This finding allowed us to compare the rGO and GO sensors and determine their elastic response depending on functional groups. The relation between responses of rGO and GO sensors can be a useful tool to discriminate NO_2_ among other interfering gases.

The response time was determined by defining τ_90_ as the time taken to reach 90% of the maximum frequency shift of the response. The MC and GO sensors presented a notable response for 0.4 ppm of NO_2_ after exposition of 120 s with a complete recovery. On the contrary, rGO and PDA/rGO sensors barely offer a noticeable response approach after NO_2_ exposition, reaching its maximum value at 80 s. (Figure 4a). All sensors showed a response for NO_2_, between 300 (sensors with sensitive layer composed of GO or MC) and 50 Hz (sensors with rGO and PDA/rGO). Furthermore, the limit of detection (LOD) achieved for NO2 was ~10 ppb on the MC sensor, ~7.5 ppb on the rGO, ~75 ppb on the GO and ~30 ppb on the PDA/rGO.

**Figure 5:**
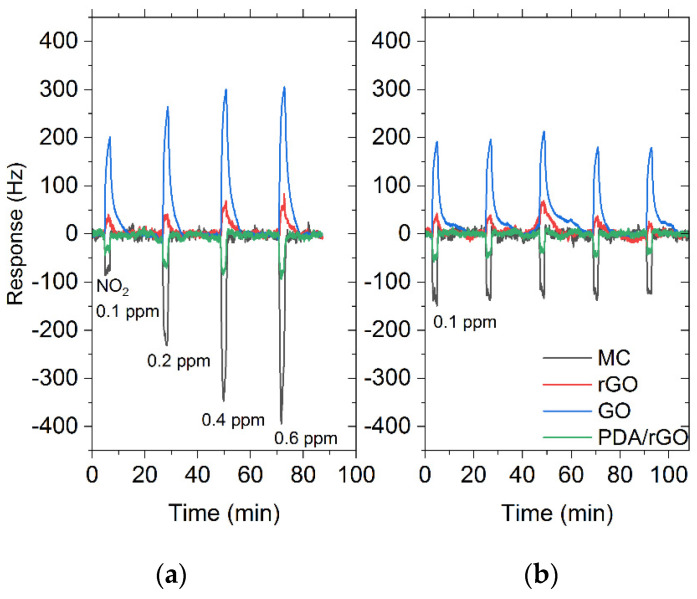
(**a**) Linearity of MC, GO, rGO and PDA/rGO obtained for different concentrations of NO_2_. (**b**) Real time response and recovery of an SH-SAW sensor with a sensitive layer for a concentration of 0.1 ppm of NO_2_.

Figure 5a shows the performance of the developed sensors for different concentration levels of NO_2_. The MC and rGO sensors achieved a high linearity response (0.98 and 0.89, respectively, adjusted R squared values) in comparison with the GO and PDA/rGO sensors (0.67 and 0.76, respectively), which shows a saturation regime behavior from 0.4 ppm of NO_2_ in advance. To verify that sensor materials are suitable as NO_2_ gas sensors we further investigate the sensors’ behavior by measuring the response in short periods (2 min) and continuous cycles of exposition and purging. Figure 5b shows the reproducibility obtained at 0.1 ppm of NO_2_. A high and stable response was obtained for MC or GO materials.

**Figure 6:**
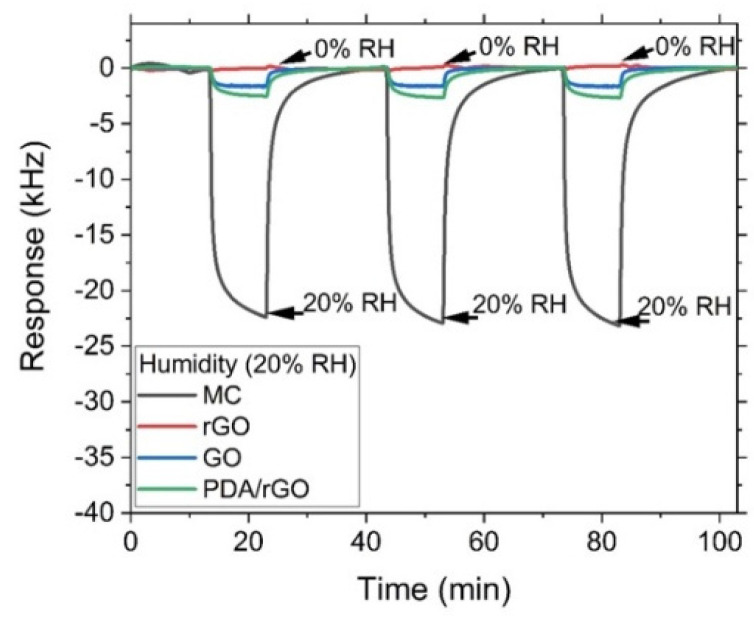
Real time response of the SH-SAW sensor array based on MC, rGO, GO and PDA/rGO materials for different humidity values at 21 °C.

The humidity influence on the sensor performance was also evaluated for discrimination capabilities. Figure 6 illustrates the sensor effect with a 20% relative humidity (RH) concentration measured with a handheld thermohygrometer (RS1364) at 21 °C. In the tested humid environment, the frequency decreased in the GO sensor, which implies a mass sensitivity mechanism. This result differs from the cases of the measured toxic gases where the frequency increased due to the elastic sensitivity mechanism (Figure 5). Accordingly, the response set from GO, rGO and PDA/GO sensors provides information valuable for distinguishing among dry and humid environments. The high detection level of sub-ppm NO_2_ makes MC and GO excellent materials with high performance for toxic gas discrimination, taking rGO and PDA/rGO as references for dry and humid environments, respectively. The negligible number of hydroxyl groups in the rGO sensitive layer implies a non-existent response to high humidity environments. However, after the rGO is covered with PDA the number of hydroxyl groups increased, presenting a high sensitivity to humidity [44,45]. For the tested humid environment, the frequency decreased in the GO sensor, which implies a mass sensitivity mechanism. This result differs from the cases of the measured toxic gases where the frequency increased due to the elastic sensitivity mechanism. Accordingly, the response set from GO, rGO and PDA/GO sensors provide information for distinguishing between dry and humid environments.

### 3.4. Statistical Treatment and Classification Analysis

**Figure 7:**
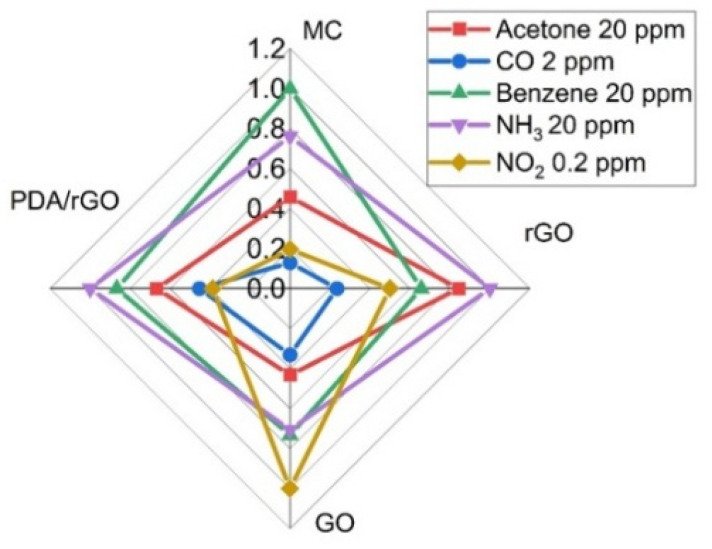
Radial representation of the sensor array’s responses to 0.2 ppm NO_2_, 2 ppm CO and 20 ppm of benzene (C_6_H_6_), acetone (C_3_H_6_O) and NH_3_.

Figure 7 shows the responses of the four SH-SAW sensors for each gas concentration, NO_2_ (0.2 ppm), CO (2 ppm), NH_3_ (20 ppm), benzene (C_6_H_6_) (20 ppm) and acetone (C_3_H_6_O) (20 ppm). In this context, the responses were normalized regarding the highest gas response for each sensor. The MC, GO, rGO sensors achieved the shortest response time for CO. Furthermore, the strong point of the elastic loading effect of the GO sensor allowed to obtain a different behavior for NO_2_ detection, facilitating NO_2_ discrimination with respect to the rest of the interfering gases.

**Figure 8:**
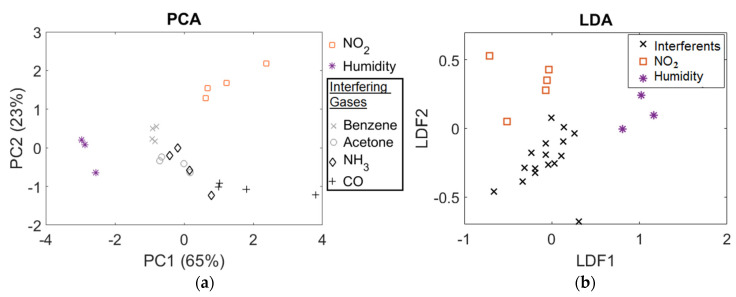
(**a**) Principal component analysis (PCA) applied to data for discrimination of interferents (black), humidity (purple) and NO_2_ (orange) for different concentrations and sensors; (**b**) LDA classification results for the three classes (NO_2_, interferents and humidity) indicates a discrimination accuracy of 90%.

We configured three ML techniques for pattern discrimination to identify sensors’ behaviors as part of different gas environments. The analysis with the unsupervised methods was focused on PCA visualization. It allowed us to investigate data dimensionality reduction and discriminate between different target gases. PCA analysis provided summarized data and visualized the information from the results containing the individual responses. It is a promising method in case of no labeled data. The scores of the two classes for the most important components—PC1 and PC2—are represented in Figure 8a, which offers a way to study the statistical discrimination and provides a cumulative contribution rate of 87%. The highest possible variation is explained by the first and second components (65% and 23%, respectively). Although low concentrations of both interfering gases, NH_3_ and acetone (C_3_H_6_O), display scattered data, PCA confirmed well-defined patterns in the sensor array response and showed a clear discrimination for NO_2_.

We also deployed two supervised ML techniques to validate the clustering of the PCA offered. This fact helped us to assure the most adequate classification method to assign the profiles to a group of responses. We conducted an (i) LDA that is a signal classification technique that directly maximizes class separability of different gases, and (ii) KNN, which is applied for classification of mixed gases providing high accuracy results. Figure 8b plots the results of the LDA projections in which the point feature set is defined by different responses. The classifier looks for the linear separability among NO_2_, interferents and humidity classes, with the examples of each class forming compact clusters and being far from each other. The result for the accuracy value (LDA classifier) was 0.9 (Figure 8b). LDA provided similar results to the PCA and, consequently, both seem to be appropriate methods for pattern discrimination in this gas sensing application.

**Figure 9:**
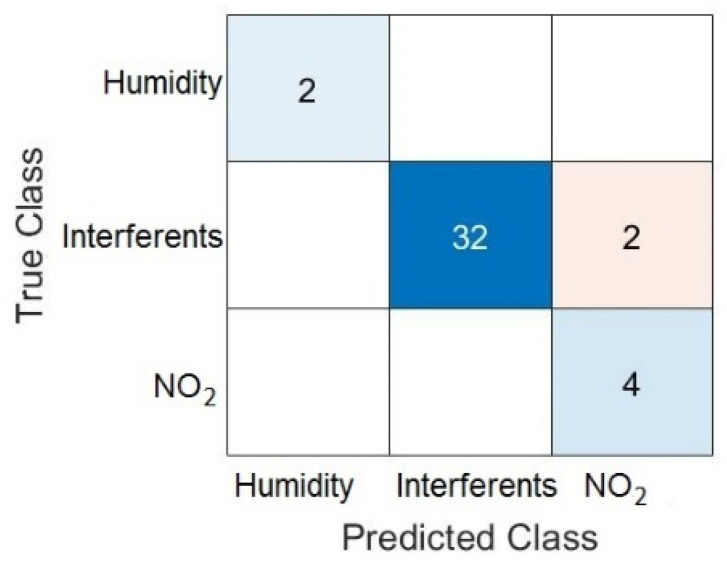
Confusion matrix obtained by KNN.

KNN is a powerful rule to generate highly nonlinear classifications with a set of data. Figure 9 shows the KNN classification performance over 38 samples from the sensors’ responses. Table 1 shows true-quality classification indices for NO_2_, interferents and humidity. The final result presents an accuracy of 0.95 for the three classes and a value up to 0.9 on both F1 and precision quality scores. The KNN classifier correctly identified NO_2_ (100%). On the contrary, two false positives were obtained for the interferents class mainly due to the heterogeneous behavior of the responses at low concentrations.

The presented study has demonstrated that a suitable sensitive layer allows measuring changes in the velocity of the SAW that would be then selectively assigned to the adsorption of the different gases. Table 2 illustrates the comparison of our developed sensitive layers with other sensing films SAW sensors reported in the literature for NOx target gas. It also shows that our materials can perform both mass and elastic detection for NO_2_ and can reach similar and improved sub-ppm detection in comparison with the previous reported SAW sensors [13,14,15,16], within less than 1 min response time. Furthermore, our SAW sensors show excellent sensitivity by mass loading effect for carbon monoxide, ammonia, acetone and benzene, which can extend the environmental gas sensing applications.

## 4. Conclusions

For the present study, we used a portable array of SH-SAW sensors for a real-time monitoring system in order to provide discrimination of toxic chemicals. The system was designed using a modular architecture, which makes it a very self-contained and versatile platform incorporating ML capabilities for gas detection such as PCA, LDA or KNN.

The e-nose successfully detected nitrogen dioxide (NO_2_), monoxide (CO), ammonia (NH_3_), benzene (C_6_H_6_) and acetone (C_3_H_6_O), in a wide variety of concentrations, with high selectivity and sensitivity, showing a pattern for each toxic agent and high efficiency to discriminate between interfering gases and NO_2_. The sensor array was effective at detecting NO_2_ even at a low concentration (0.1 ppm). On the other hand, the elastic-loading effect on the GO-based sensor shows a characteristic behavior for NO_2_ which, together with the other sensors, allows NO_2_ to be discriminated from other interferents. The PCA plot data clustering did not present a significant discrimination between the interfering gases, but it showed that NO_2_ was discriminated from these other gases. Thus, PCA did not provide a clear discrimination among interfering gases with high selectivity and sensitivity, mainly due to the similar responses of acetone or ammonia at low concentrations. The SH-SAW sensors showed different sensitivities at room temperature, excellent repeatability and fast responses, and therefore all of them are considered good candidates for compact array sensors (e-nose) for environmental applications.

## Figures and Tables

**Table 1:** Sensitivity, F1 and precision scores for KNN classification (60% tested out of the total sample).

	Precision	Sensitivity	F1-Score
Humidity	1	0.94	0.97
Interferents	0.67	1	0.8
NO_2_	1	1	1
Accuracy		0.95%	

**Table 2:** Comparison of different SAW sensor parameters reported in the literature for NO_x_ target gas.

Sensitive Layer	Target Gas	Operating Frequency	Sensitivity	Detected ppm	Response Time	Detection Mechanics	Reference
SnO_2_	NO_2_	433.9 Mhz	-	20 ppm	2 s	Elastic loading	[13]
ZnO	NO_2_	99.5 Mhz	2.9 Hz/ppb	400 ppb–16 ppm	-	Mass loading	[14]
PPy/WO/rGO	NO	98 Hhz	12 Hz/ppb	5–110 ppb	<2min	Mass loading	[15]
PZT	NO_2_	99.4 Mhz	9.6 Hz/ppm	80–250 ppm	-	Mass loading	[16]
MC rGO GO PDA/rGO	NO_2_	160 Mhz	10 Hz/ppb 7.5 Hz/ppb 75 Hz/ppb 30 Hz/ppb	0.1–1 ppm	<1min	Mass loading Elastic loading Elastic loading Mass loading	This work

## Data Availability

Not applicable.

## Abbreviations

PPy: polypyrrole; PZT: lead zirconate titanate.

## References

[1] Tan X., Han L., Zhang X., Zhou W., Li W., Qian Y. (2021). A review of current air quality indexes and improvements under the multi-contaminant air pollution exposure. J. Environ. Manag..

[2] Chen T.M., Kuschner W.G., Gokhale J., Shofer S. (2007). Outdoor air pollution: Nitrogen dioxide, sulfur dioxide, and carbon mon-oxide health effects. Am. J. Med. Sci..

[3] Wang C., Yin L., Zhang L., Xiang D., Gao R. (2010). Metal Oxide Gas Sensors: Sensitivity and Influencing Factors. Sensors.

[4] Han X., Naeher L.P. (2006). A review of traffic-related air pollution exposure assessment studies in the developing world. Environ. Int..

[5] SM S.N., Yasa P.R., Narayana M.V., Khadirnaikar S., Rani P. (2019). Mobile monitoring of air pollution using low-cost sen-sors to visualize spatio-temporal variation of pollutants at urban hotspots. Sustain. Cities Soc..

[6] Covington J.A., Marco S., Persaud K.C., Schiffman S.S., Nagle H.T. (2021). Artificial Olfaction in the 21st Century. IEEE Sens. J..

[7] Shaw C., Boulic M., Longley I., Mitchell T., Pierse N., Howden-Chapman P. (2020). The association between indoor and outdoor NO_2_ levels: A case study in 50 residences in an urban neighbourhood in New Zealand. Sustain. Cities Soc..

[8] Shea K.M., Truckner R.T., Weber R.W., Peden D. (2008). Climate change and allergic disease. J. Allergy Clin. Immunol..

[9] Bahos F.A., Sainz-Vidal A., Sánchez-Pérez C., Saniger J.M., Gràcia I., Saniger-Alba M.M., Matatagui D. (2018). ZIF Nanocrystal-Based Surface Acoustic Wave (SAW) Electronic Nose to Detect Diabetes in Human Breath. Biosensors.

[10] Vanotti M., Poisson S., Soumann V., Quesneau V., Brandès S., Desbois N., Yang J., André L., Gros C.P., Blondeau-Patissier V. (2021). Influence of interfering gases on a carbon monoxide differential sensor based on SAW devices functionalized with cobalt and copper corroles. Sens. Actuators B Chem..

[11] Zhu H., Xie D., Lin S., Zhang W., Yang Y., Zhang R., Shi X., Wang H., Zhang Z., Zu X. (2021). Elastic loading enhanced NH_3_ sensing for surface acoustic wave sensor with highly porous nitrogen doped diamond like carbon film. Sens. Actuators B Chem..

[12] Matatagui D., López-Sánchez J., Peña A., Serrano A., del Campo A., de la Fuente O.R., Carmona N., Navarro E., Marín P., Horrillo M.D.C. (2021). Ultrasensitive NO_2_ gas sensor with insignificant NH_3_-interference based on a few-layered mesoporous graphene. Sens. Actuators B Chem..

[13] Raj V.B., Nimal A.T., Tomar M., Sharma M.U., Gupta V. (2015). Novel scheme to improve SnO_2_/SAW sensor performance for NO_2_ gas by detuning the sensor oscillator frequency. Sens. Actuators B Chem..

[14] Rana L., Gupta R., Tomar M., Gupta V. (2017). ZnO/ST-Quartz SAW resonator: An efficient NO_2_ gas sensor. Sens. Actuators B Chem..

[15] Hung T.-T., Chung M.-H., Chiu J.-J., Yang M.-W., Tien T.-N., Shen C.-Y. (2021). Poly(4-styrenesulfonic acid) doped polypyrrole/tungsten oxide/reduced graphene oxide nanocomposite films based surface acoustic wave sensors for NO sensing behavior. Org. Electron..

[16] Rana L., Gupta R., Kshetrimayum R., Tomar M., Gupta V. (2018). Fabrication of surface acoustic wave based wireless NO_2_ gas sensor. Surf. Coat. Technol..

[17] Ghosh A., Zhang C., Shi S., Zhang H. (2019). High temperature CO_2_ sensing and its cross-sensitivity towards H_2_ and CO gas using calcium doped ZnO thin film coated langasite SAW sensor. Sens. Actuators B Chem..

[18] Sayago I., Matatagui D., Fernández M.J., Fontecha J.L., Jurewicz I., Garriga R., Muñoz E. (2016). Graphene oxide as sensitive layer in Love-wave surface acoustic wave sensors for the detection of chemical warfare agent simulants. Talanta.

[19] Matatagui D., Fernández M., Fontecha J., Sayago I., Gràcia I., Cane C., Horrillo C., Santos J. (2014). Characterization of an array of Love-wave gas sensors developed using electrospinning technique to deposit nanofibers as sensitive layers. Talanta.

[20] Fragoso-Mora J., Matatagui D., Bahos F., Fontecha J., Fernandez M., Santos J., Sayago I., Gràcia I., Horrillo M. (2018). Gas sensors based on elasticity changes of nanoparticle layers. Sens. Actuators B Chem..

[21] Matatagui D., Fernández M., Fontecha J., Santos J., Gràcia I., Cane C., Horrillo C. (2015). Propagation of acoustic waves in metal oxide nanoparticle layers with catalytic metals for selective gas detection. Sens. Actuators B Chem..

[22] Raj V.B., Nimal A.T., Parmar Y., Sharma M.U., Gupta V. (2012). Investigations on the origin of mass and elastic loading in the time varying distinct response of ZnO SAW ammonia sensor. Sens. Actuators B Chem..

[23] Raj V.B., Singh H., Nimal A., Tomar M., Sharma M., Gupta V. (2013). Effect of metal oxide sensing layers on the distinct detection of ammonia using surface acoustic wave (SAW) sensors. Sens. Actuators B Chem..

[24] Matatagui D., Bahos F.A., Gràcia I., Horrillo M.D.C. (2019). Portable Low-Cost Electronic Nose Based on Surface Acoustic Wave Sensors for the Detection of BTX Vapors in Air. Sensors.

[25] Park S.Y., Kim Y., Kim T., Eom T.H., Kim S.Y., Jang H.W. (2019). Chemoresistive materials for electronic nose: Progress, perspectives, and challenges. InfoMat.

[26] El Kazzy M., Weerakkody J.S., Hurot C., Mathey R., Buhot A., Scaramozzino N., Hou Y. (2021). An overview of artificial olfaction systems with a focus on surface plasmon resonance for the analysis of volatile organic compounds. Biosensors.

[27] Raj V.B., Singh H., Nimal A.T., Sharma M.U., Tomar M., Gupta V. (2017). Distinct detection of liquor ammonia by ZnO/SAW sensor: Study of complete sensing mechanism. Sens. Actuators B Chem..

[28] Tang Y., Xu X., Han S., Cai C., Du H., Zhu H., Zu X., Fu Y. (2020). ZnO-Al_2_O_3_ nanocomposite as a sensitive layer for high performance surface acoustic wave H2S gas sensor with enhanced elastic loading effect. Sens. Actuators B Chem..

[29] Cruz C., Palomar E., Bravo I., Aleixandre M. (2021). Behavioural patterns in aggregated demand response developments for commu-nities targeting renewables. Sustain. Cities Soci..

[30] Marczyński P., Szpakowski A., Tyszkiewicz C., Pustelny T. (2012). Pattern Recognition Applied to Analysis of Gas Sensors’ Array Data. Acta Phys. Pol. A.

[31] Jha S.K., Yadava R.D.S. (2009). Preprocessing of SAW Sensor Array Data and Pattern Recognition. IEEE Sens. J..

[32] Singh H., Raj V.B., Kumar J., Durani F., Mishra M., Nimal A.T., Sharma M.U. (2016). SAW mono sen-sor for identification of harmful vapors using PCA and ANN. Process. Saf. Environ. Prot..

[33] García M., Fernández M., Fontecha J., Lozano J., Santos J., Aleixandre M., Sayago I., Gutiérrez J., Horrillo M. (2006). Differentiation of red wines using an electronic nose based on surface acoustic wave devices. Talanta.

[34] Herrera-Chacon A., Campos I., González-Calabuig A., Torres M., del Valle M. (2021). Coupling of Sensors and Machine Learning Algorithms in the Qualitative Analysis of Wine. Eng. Proc..

[35] Ciosek P., Wróblewski W. (2006). The analysis of sensor array data with various pattern recognition techniques. Sens. Actuators B Chem..

[36] Zhang W., Tian F., Song A., Hu Y. (2018). Research on electronic nose system based on continuous wide spectral gas sensing. Microchem. J..

[37] Dang L., Tian F., Zhang L., Kadri C., Yin X., Peng X., Liu S. (2014). A novel classifier ensemble for recognition of multiple indoor air contaminants by an electronic nose. Sens. Actuators A Phys..

[38] Sisk B.C., Lewis N.S. (2003). Estimation of chemical and physical characteristics of analyte vapors through analysis of the response data of arrays of polymer-carbon black composite vapor detectors. Sens. Actuators B Chem..

[39] Feng S., Farha F., Li Q., Wan Y., Xu Y., Zhang T., Ning H. (2019). Review on Smart Gas Sensing Technology. Sensors.

[40] Al-Dayyeni W.S., Al-Yousif S., Taher M.M., Al-Faouri A.W., Tahir N.M., Jaber M.M., Ghabban F., Najm I.A., Alfadli I.M., Ameerbakhsh O.Z. (2021). A Review on Electronic Nose: Coherent Taxonomy, Classification, Motivations, Challenges, Recommendations and Datasets. IEEE Access.

[41] Gutiérrez J., Horrillo M.C. (2014). Advances in artificial olfaction. Sens. Appl. Talanta.

[42] de la O-Cuevas E., Alvarez-Venicio V., Badillo-Ramírez I., Islas S.R., Carreón-Castro M.D.P., Saniger J.M. (2020). Graphenic substrates as modifiers of the emission and vibrational responses of interacting molecules: The case of BODIPY dyes. Spectrochim. Acta Part A Mol. Biomol. Spectrosc..

[43] Lu G., Ocola L.E., Chen J. (2009). Reduced graphene oxide for room-temperature gas sen-sors. Nanotechnology.

[44] Zhang D., Song X., Wang Z., Chen H. (2021). Ultra-highly sensitive humidity sensing by polydopa-mine/graphene oxide nanostructure on quartz crystal microbalance. Appl. Surface Sci..

[45] Hallil H., Zhang Q., Flahaut E., Lachaud J.-L., Coquet P., Dejous C., Rebière D. (2018). Guided SH-SAW sensor based on DWNTs sensitive material for VOCs and humidity detection. J. Integr. Circuits Syst..

